# Sleep, physical activity, and sedentary behaviors as factors related to depression and health-related quality of life among older women living alone: a population-based study

**DOI:** 10.1186/s11556-023-00314-7

**Published:** 2023-03-13

**Authors:** Soyoung Jang, Eunjin Yang

**Affiliations:** 1 Department of Nursing, Kyungil University, Gyeongsan, Republic of Korea; 2 Mo-Im Kim Nursing Research Institute, Yonsei University College of Nursing, Seoul, Republic of Korea; 3 College of Nursing, Gachon University, 191 Hambangmoe-Ro, Yeonsu-Gu, Incheon, 21936 Republic of Korea

**Keywords:** Older adults, Depression, Quality of life, Physical activity, Sedentary behavior, Sleep

## Abstract

**Background:**

Although the number of older women living alone (OWLA) has risen steadily in aging societies, and research has been conducted on depression and health-related quality of life (HRQoL) among older adults, research is scarce on the health behaviors of OWLA, including their sleep, physical activity, and sedentary behaviors. Hence, we aimed to identify factors related to depression and HRQoL among this subset of the population, focusing on their health behaviors, using Andersen’s model as a research framework.

**Methods:**

Data for secondary analysis were from the Korean National Health and Nutritional Examination Survey (2014, 2016, 2018, and 2020). The inclusion criteria were (1) women aged 65 and older and (2) those living alone. We included 794 older South Korean women living alone from 31,051 respondents. We used hierarchical regression analysis, considering sampling weight and a complex sample design, to identify factors related to depression and HRQoL.

**Results:**

Among the health behavior factors of Andersen’s model as a research framework, sleep was associated with depression, whereas physical activity and sedentary behaviors were related to HRQoL. Subjective health status, limited activity, and perceived stress were associated with both depression and HRQoL. Household income, as an enabling factor, was only associated with HRQoL. The final regression model explained 39% of the variance in depression (*p* < 0.001) and 37% of the variance in HRQoL (*p* < 0.001).

**Conclusions:**

Our study highlights the importance of strategies to improve specific healthy behaviors that affect depression and HRQoL in OWLA. Appropriate interventions that target increasing physical activity and quality of sleep, and decreasing sedentary behaviors, will be effective to enhance the well-being of OWLA. Healthcare providers should comprehensively understand the characteristics of OWLA and pay more attention to enabling, need, and health behavior factors.

**Supplementary Information:**

The online version contains supplementary material available at 10.1186/s11556-023-00314-7.

## Background

The World Health Organization (WHO) predicted that the number of adults aged 60 and older would nearly double from 1 to 2.1 billion between 2020 and 2050 [1]. Along with the rapidly growing aging population, the proportion of adults aged 65 and older in South Korea is expected to rise from 15.7% in 2020 to 46.4% by 2070 [2]. Furthermore, the number of older adults living alone is steadily expanding, and 71.9% of older adults living alone are women, whereas approximately 28.1% are men [3]. This growing subset of the wider population and the number of single-person households are global trends, which might be due to household diversity according to increased life expectancy, nuclear families, and changes in family structure and values [4].

Older adults who live alone are affected by economic vulnerability and physical and mental health problems [5]. They have more chronic diseases, depression, social isolation, and a lower quality of life than do older adults who live with others [6, 7]. In particular, older women living alone (OWLA) have poorer health and economic status than older men living alone; this vulnerability increases stress and depression among OWLA [8]. Therefore, OWLA have greater rates of depression than their male peers [9], and their high level of depression increases their risk of suicide [10]. Given the vulnerability of OWLA and their high rate of depression, depression deserves more research attention as a mental health priority for this sector of the broader population.

Health-related quality of life (HRQoL) is a vital factor of healthy aging [11] and refers to “how well a person functions in their life and perceived wellbeing in physical, mental, and social domains of health” [12]. Thus, it offers a multidimensional perspective that includes physical, social, and psychological functions. The quality of life of older adults living alone is lower than that of the general adult population; further, among persons who live alone, women have a lower quality of life than do men [13]. With the growing number of OWLA, identifying factors related to HRQoL and maintaining HRQoL by facilitating such factors are priorities in health policy for healthcare providers and policymakers.

Previous studies have reported factors that affect depression and quality of life in older adults living alone. Participation in social and religious gatherings, level of physical activities and leisure activities, and subjective low economic status are associated with depression in this population [14–16]. Furthermore, cognitive function, suicidal thoughts, and depressive symptoms have been identified as factors related to HRQoL in older adults living alone [13]. Although depression and HRQoL are linked to older adults’ multidimensional characteristics, and some have already been revealed, few studies rooted in a theoretical foundation have been conducted on the general factors tied to depression and HRQoL. Moreover, research on women and healthy behaviors regarding depression and HRQoL is limited. Hence, we aimed to identify factors associated with depression and HRQoL in OWLA based on Andersen’s model, with a focus on sleep, physical activity, and sedentary behaviors.

### Conceptual framework

We adopted Andersen’s model as a conceptual framework to ensure research rigor when predicting factors related to depression and HRQoL. Andersen’s original model has been frequently used to explain healthcare service use or unmet healthcare needs through several components such as predisposing, enabling, and need factors [17]. The most recent version of the model added health behavior factors to explain not only medical service use and consumer satisfaction, but also health outcomes such as quality of life [18]. Therefore, Andersen’s is an integrated model that predicts health-related outcomes considering individual, contextual, and health behaviors characteristics [19]. Notably, the model is helpful for planning practical interventions to prevent negative health outcomes and manage older adults’ health conditions.

Predisposing factors refer to demographic traits and social structures such as gender, age, marital status, education level, and social class [18, 20]. Enabling factors, also known as enabling resources, refer to methods or knowledge that makes it possible or impossible for individuals to access medical services within the context of their families and communities [18]. Such resources encompass health insurance, income, activities of daily living (ADLs), residential areas, and the price of health services. Need factors directly cause individuals to use medical services, such as subjective health status (SHS), disability, and chronic diseases [20, 21]. Finally, health behaviors denotes health-related activities associated with an individual’s health outcomes, including physical activities, leisure activities, self-care, smoking, and alcohol consumption [18, 19]. Studies of quality of life among older adults have been conducted by applying Andersen’s model as a research framework [22–24]. However, research on depression and HRQoL in OWLA using this model is scarce, especially research focusing on health behaviors.

## Methods

### Study design and participants

**Fig. 1:**
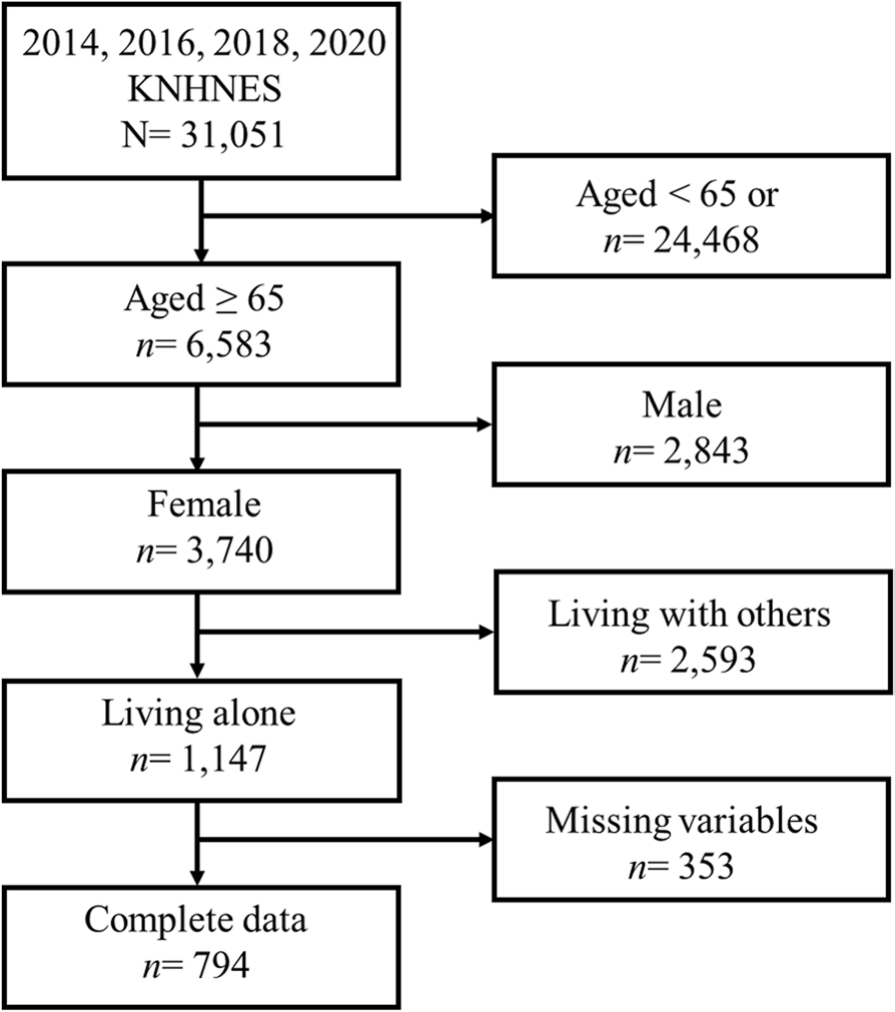
Flow diagram of participants selection

We performed secondary data analysis and employed data from the 2014, 2016, 2018, and 2020 Korea National Health and Nutrition Examination Survey (KNHANES), which is an ongoing survey. The KNHANES is a nationally representative, cross-sectional survey of health status, health behaviors, food, and nutrition. The reason for selecting data from specific years (2014, 2016, 2018, and 2020) was that depression—an essential variable in this study—was assessed during these years. We obtained the data from the Korea Disease Control and Prevention Agency; researchers can download de-identified data from the website without any specific process to request information. This survey used multistage stratified cluster sampling. Once districts for the KNHANES were selected from the districts for the “National Population and Housing Census,” households eligible for the survey were selected after checking the household members. There was little possibility that the same participants were selected in consecutive waves because double-checking was conducted when selecting survey districts and households, even though sampling was performed separately in every survey. A total of 31,051 participants took part in the KNHANES in the years we scrutinized (2014, 2016, 2018, 2020); we excluded participants younger than 65, those who were male, those living with others, and those with at least one missing data element of the variables assessed in this study. Approximately 30.8% of the unweighted sample was excluded because of missing data. Detailed information is provided in supplemental Table 1. We included a total of 794 OWLA; the detailed process of participant selection is depicted in Fig. 1.

### Variables

#### Predisposing factors

Predisposing factors refer to inherent socio-demographic characteristics such as age, gender, and marital status [21]. We included age and education level in this study as predisposing factors. All questionnaires for the variables included in this study variables are presented in supplemental Table 2.

#### Enabling factors

Enabling factors refer to organizational and financial characteristics that allow people to use medical services and achieve health outcomes [21]. We included equivalent income and economic activity as enabling factors. Equivalized income was represented as a numerical variable according to the population’s calculated household income quintile: low, lower-middle, middle, upper-middle, and high. Economic activity status was assessed using the question, “Have you ever done paid work for one hour or more, or unpaid work for more than 18 h, in the past week?” The answers were either yes or no.

#### Need factors

Need factors are physiological and psychological factors related to disability or health that cause an individual to require medical services; they include subjective or objective health status [20]. Need factors are more closely tied to health outcomes or the need to use medical services compared to other factors, such as predisposing and enabling factors. We selected variables such as multimorbidity, subjective health status, activity limitation, and perceived stress as need factors. In the present study, multimorbidity refers to the number of chronic diseases that currently have among 31 chronic illnesses, including hypertension, dyslipidemia, ischemic heart disease, stroke, diabetes, thyroid disease, cancer, hepatitis B or C, osteoporosis, osteoarthritis, pulmonary tuberculosis, and asthma. To assess SHS, participants were asked to rate their health on a 5-point scale: (1) very poor, (2) poor, (3) fair, (4) good, and (5) very good. Activity limitation was assessed using a single question: “Do you currently have restrictions in daily life or social activities because of health problems or a physical or mental disability?” The answers were yes or no. Perceived stress was evaluated using a single question: “Are you feeling stressed out in daily life?” Participants rated perceived stress on a 4-point scale (1 = little, 2 = a little bit, 3 = a lot, 4 = very much); thus, a high score indicated a high level of stress.

#### Health behaviors

Sleep, physical activity, and sedentary behaviors as health behaviors were assessed using a self-reported questionnaire. Participants were asked about their average sleep duration on weekdays and weekends, and the average sleep duration per day was calculated [25–27]. In the present study, physical activity and sedentary behaviors were assessed using the Global Physical Activity Questionnaire (GPAQ) [28], developed by the World Health Organization, which is a reliable and valid tool to assess physical activity. The tool collects information regarding moderate-to-vigorous physical activity involved in work, transportation, and recreation. Two variables, assessed in MET minutes per week, representing moderate-intensity physical activity and vigorous-intensity physical activity as continuous variables were generated according to the GPAQ analysis guidelines. Sedentary behavior was also assessed using the GPAQ questionnaire; respondents were asked about the average time in minutes spent sitting or lying down per day, except for sleep.

#### Depression

The Patient Health Questionaire-9 (PHQ-9) is a self-report questionnaire developed to promote the diagnosis of mental illness in primary care [29]. The PHQ-9 is a reliable and valid tool consisting of 9 items, each of which is rated on a 4-point Likert scale. The items ask how frequently specific symptoms occur to diagnose a depressive disorder, ranging from 0 to 3 (0 = not at all, 1 = several days, 2 = more than half of the days, 3 = nearly every day). A high score indicates a high level of depressive symptoms, with scores ranging from 0 to 27. Cronbach’s alpha is 0.81, and test–retest reliability is 0.89 [30]; therefore, the reliability of the Korean version of the PHQ-9 has been supported. Concurrent validity of the instrument was verified in a previous study [30].

#### Health-related quality of life

HRQoL was measured using the EuroQol 5-Dimension (EQ5D), which is a standardized instrument. The EQ5D is divided into five domains (mobility, usual activity, self-care, pain/discomfort, and anxiety/depression). Responses are rated on a scale of 1 to 3, with 1 indicating no problem, 2 denoting some problems, and 3 implying extreme problems [31]. The EQ5D score was calculated as a single number between 0 (as bad as being dead) and 1 (full health) through weights for health status, with a higher score signaling better quality of life. We used the EQ5D index; hence, we converted all answers to an index using weights developed based on the South Korean population [32]. The reliability and validity of the Korean version of the EQ5D have been demonstrated in prior studies [33].

### Ethical considerations

The Institutional Review Board (IRB) of the Korea Disease Control and Prevention Agency reviewed and approved the 2014 (2013-12EXP-03-5C), 2018 (2018–01-03-P-A), and 2020 (018–01-03-2C-A) surveys, whereas the 2016 survey was not reviewed by the IRB to obtain ethical permission. However, the survey was initiated by the government, and the Bioethics and Safety Act guarantees this survey is for the public good, regardless of IRB permission. All participants received detailed information prior to being involved in this national survey, including the survey purposes, process, and the possibility of their anonymized data being provided to other researchers. Informed consent was obtained from all participants and researchers can access the anonymized KNHANES dataset without further request. Therefore, this secondary data analysis study was exempted from review by the board of ethics at the author’s affiliated institution.

### Statistical analysis

Multistage stratified cluster sampling was used to ensure the characteristics of a nationally representative sample. Thus, 4-year sample weights were computed for complex sample analysis according to the guidelines of KNHANES 8, developed by the Korea Disease Control and Prevention Agency [34]. Characteristics of complex sampling—such as strata, clusters, and sampling weights—were considered throughout the analytical process. Descriptive analysis to present the participants’ characteristics was conducted such that continuous variables were expressed as means and standard errors, and categorical variables were presented as unweighted numbers and weighted counts. Complex-sample hierarchical regression analysis was performed to verify the association between factors based on Andersen’s model and depression. We also investigated the relationship between these factors and HRQoL among OWLA. According to Andersen’s model, we entered factors as blocks such as predisposing factors, enabling factors, need factors, and health behaviors factors in the regression model, step-by-step. We did not find any multicollinearity through checking the variance inflation factor (VIF, 1.01–1.64). Significance was defined as *p* < 0.05. We performed all statistical analyses using SPSS software, Version 26 (IBM Corp., Armonk, NY, US).

## Results

### General characteristics of the participants

The participants’ mean age was 74.8 years and more than two-thirds had an education level of less than elementary school. The largest proportion (c. 92.4%) had a lower or lower-middle economic status, and 27.3% had an economically active status. Regarding health status, OWLA had an average of 2.6 chronic diseases and c. 22.9% had limited activity. The participants’ average sleep duration was 387.6 min/day (6.5 h) and vigorous and moderate energy expenditure were 11.1 Met-minutes/week and 441.5 Met-minutes/week, respectively. Average sedentary time was 566.6 min/day (9.4 h/day). The level of depression investigated using the PHQ-9 was 3.7, and the EQ5D index representing HRQoL was 0.8 among OWLA (Table 1).

**Table 1:** General characteristics of participants (*N* = 794)

Variables	Mean ± SE or N(%)
Depression (PHQ-9)	3.7 ± 0.2
Health-related quality of life (EQ-5D)	0.8 ± 0.0
***Predisposing factors***	
Age	74.8 ± 0.2
Education	
Below elementary	633 (78.4)
Middle school	89 (11.6)
High school and above	72 (10.0)
***Enabling factors***	
Equivalised income	
Lower	575 (71.4)
Lower middle	161 (21.0)
Middle	37 (4.7)
Upper middle	16 (2.2)
Upper	5 (0.7)
Economic activity	
Economically active	227 (27.3)
Economically inactive	567 (72.7)
***Need factors***	
Multimorbidity	2.6 ± 0.1
Subjective health status	2.6 ± 0.0
Activity limitation	
Yes	186 (22.9)
No	608 (77.1)
Perceived stress	1.8 ± 0.0
***Health behaviors***	
Sleep (mins/day)	387.6 ± 4.3
Vigorous physical activity (MET-minutes/week)	11.1 ± 4.4
Moderate physical activity (MET-minutes/week)	441.5 ± 35.6
Sedentary behaviors (mins/day)	566.6 ± 9.7

N (%), unweighted count (weighted %); M ± SE, estimated mean ± standard error

### Factors associated with the depression and health-related qualify of life

We entered a total of 12 independent variables into the hierarchical regression model. Tables 2 and 3 display the results of the model to verify factors related to depression and HRQoL. The R^2^ of predisposing factors was 1% in the depression model I (F = 3.78, *p* = 0.01) and an additional 2% of variance was explained after introducing enabling factors. Need factors including multimorbidity, subjective health status, activity limitation, and stress explained a large proportion of the variance in depression (34%). The R^2^ value of the final depression model was 0.39 (i.e., 39% of variance explained; F = 26.50, *p* < 0.001), which increased by 0.2% after entering health behaviors. Subjective health status (*p* < 0.001), activity limitation (*p* < 0.001), stress (*p* < 0.001), and sleep (*p* < 0.001) were statistically significant factors in the final model (Table 2).

**Table 2:** Hierarchical multiple regression prediction of depression (*N* = 794)

	**Model 1**		**Model 2**		**Model 3**		**Model 4**	
	**B(SE)**	**t(p)**	**B(SE)**	**t(p)**	**B(SE)**	**t(p)**	**B(SE)**	**t(p)**
**(Constant)**	3.89 (3.03)	1.28 (0.20)	8.97 (3.72)	2.41 (0.02)	3.03 (2.80)	1.08 (0.28)	5.68 (2.89)	1.96 (0.05)
**Predisposing factors**								
Age	-0.02 (0.04)	-0.41 (0.68)	-0.06 (0.05)	-3.46 (< 0.001)	-0.01 (0.03)	-0.23 (0.82)	-0.02 (0.03)	-0.53 (0.59)
Education (Reference = high school and above)								
Below elementary	1.40 (0.63)	2.23 (0.03)	0.90 (0.67)	1.33 (0.18)	0.09 (0.60)	0.16 (0.88)	0.02 (0.59)	0.03 (0.97)
Middle school	0.04 (0.70)	0.05 (0.96)	-0.24 (0.73)	-0.33 (0.74)	-0.09 (0.64)	-0.14 (0.89)	-0.20 (0.63)	-0.32 (0.75)
**Enabling factors**								
Equivalised income			-0.86 (0.25)	-3.46 (< 0.001)	-0.35 (0.22)	-1.63 (0.10)	-0.38 (0.21)	-1.80 (0.07)
Economic activity (Reference = Inactive)			-0.82 (0.43)	-1.92 (0.06)	-0.59 (0.33)	-1.78 (0.08)	-0.46 (0.34)	-1.35 (0.18)
**Need factors**								
Multimorbidity					0.16 (0.12)	1.33 (0.18)	0.15 (0.12)	1.24 (0.22)
Subjective health status					-1.18 (0.21)	-5.65 (< 0.001)	-1.12 (0.20)	-5.64 (< 0.001)
Activity limitation (Reference = No)					2.47 (0.45)	5.43 (< 0.001)	2.48 (0.46)	5.45 (< 0.001)
Perceived stress					2.25 (0.24)	9.43 (< 0.001)	2.20 (0.23)	9.48 (< 0.001)
**Health behaviors**								
Sleep (mins/day)							-0.01 (0.00)	-3.73 (< 0.001)
Vigorous physical activity (MET-minutes/week)							0.00 (0.00)	0.85 (0.39)
Moderate physical activity (MET-minutes/week)							0.00 (0.00)	0.57 (0.57)
Sedentary behaviors (mins/day)							0.00 (0.00)	1.55 (0.12)
**R**^**2**^ ** (△R**^**2**^**)**	0.01		0.03 (0.02)***		0.37 (0.34)***		0.39 (0.02)***	
**F (P)**	3.78 (0.01)		6.18 (< 0.001)		32.73 (< 0.001)		26.50 (< 0.001)

**Table 3:** Hierarchical multiple regression prediction of health-related quality of life (*N* = 794)

	**Model 1**		**Model 2**		**Model 3**		**Model 4**	
	**B(SE)**	**t(p)**	**B(SE)**	**t(p)**	**B(SE)**	**t(p)**	**B(SE)**	**t(p)**
**(Constant)**	1.29 (0.14)	9.44 (< 0.001)	1.03 (0.15)	7.06 (< 0.001)	1.02 (0.12)	8.84 (< 0.001)	0.98 (0.11)	8.85 (< 0.001)
**Predisposing factors**								
Age	-0.01 (0.00)	-2.89 (< 0.001)	0.00 (< 0.001)	-1.63 (0.10)	0.00 (0.00)	-2.56 (0.01)	0.00 (0.00)	-1.69 (0.09)
Education (Reference = high school and above)								
Below elementary	-0.07 (0.02)	-3.80 (< 0.001)	-0.05 (0.02)	-2.57 (0.01)	-0.01 (0.01)	-0.99 (0.32)	-0.02 (0.01)	-1.15 (0.25)
Middle school	0.01 (0.02)	0.36 (0.72)	0.02 (0.02)	1.01 (0.31)	0.02 (0.02)	1.24 (0.21)	0.01 (0.02)	-0.84 (0.40)
**Enabling factors**								
Equivalised income			0.04 (0.01)	4.89 (< 0.001)	0.02 (0.01)	2.33 (0.02)	0.02 (0.01)	2.39 (0.02)
Economic activity (Reference = Inactive)			0.05 (0.02)	3.03 (< 0.001)	0.03 (0.01)	2.31 (0.02)	0.02 (0.01)	1.65 (0.10)
**Need factors**								
Multimorbidity					-0.01 (0.01)	-1.31 (0.19)	0.00 (0.01)	-0.68 (0.50)
Subjective health status					0.07 (0.01)	8.68 (< 0.001)	0.07 (0.01)	8.42 (< 0.001)
Activity limitation (Reference = No)					-0.09 (0.02)	-4.64 (< 0.001)	-0.08 (0.02)	-4.10 (< 0.001)
Perceived stress					-0.03 (0.01)	-2.99 (< 0.001)	-0.03 (0.01)	-3.11 (< 0.001)
**Health behaviors**								
Sleep (mins/day)							0.00 (0.00)	-0.20 (0.84)
Vigorous physical activity (MET-minutes/week)							0.00 (0.00)	-6.36 (< 0.001)
Moderate physical activity (MET-minutes/week)							0.00 (0.00)	2.46 (0.01)
Sedentary behaviors (mins/day)							0.00 (0.00)	-4.34 (< 0.001)
**R**^**2**^** (△R**^**2**^**)**	0.05		0.08 (0.03)***		0.35 (0.27)***		0.37 (0.02)***	
**F (P)**	14.62 (< 0.001)		15.74(< 0.001)		26.05 (< 0.001)		23.12 (< 0.001)	

^***^ *p* < 0.001

Predisposing and enabling factors explained 5% and 3% of the variance in HRQoL in model I and II, respectively. Need factors including multimorbidity, subjective health status, activity limitation, and stress explained an additional 27% of the variance in depression (F = 26.50, *p* < 0.001). Finally, health behaviors explained 2% of the variance in HRQoL in the final model. Vigorous physical activity (*p* < 0.001), moderate physical activity (*p* = 0.01), and sedentary behaviors (*p* < 0.001) among health behaviors were statistically significant in the final HRQoL model. In addition, equivalized income (*p* = 0.02) among enabling factors and subjective health status (*p* < 0.001), activity limitation (*p* < 0.001), and stress (*p* < 0.001) among need factors were significantly associated with HRQoL. The final model explained 37% of the variance in HRQoL (Table 3).

Additional information pertaining to two separate multivariate linear regression models that included only health behaviors factors as independent variables to show the association between health behaviors factors and both depression and health-related quality of life; is presented in supplemental Table 3.

## Discussion

This study identified factors related to depression and HRQoL in OWLA using nationally representative data from four years of KNHANES (2014, 2016, 2018, and 2020). Furthermore, we tried to comprehensively understand depression and HRQoL in OWLA using a research framework based on Andersen’s model. As the results, need factors and health behaviors among four components of Andersen’s model were closely associated with depression and HRQoL.

The level of depression was 3.7, and the EQ5D index was 0.8 for OWLA. Depression among OWLA in this study was higher than that in older adults living with children or living as a couple [35] and lower than that among older adults with chronic musculoskeletal pain [36]. The HRQoL of OWLA was lower than that of persons with multiple chronic conditions [37] and comparable to that of persons with disabilities in South Korea [38]. That is, OWLA had a somewhat lower degree of depression than do people with specific health conditions such as chronic pain, and the HRQoL of OWLA was worse than those with poor health status, such as chronic ailments. Given the poor mental health, including loneliness and depression, and poor HRQoL of OWLA, more attention and theory-driven approaches are needed to reduce depression and improve HRQoL in this population.

Similar to previous studies, among health behaviors, sleep was associated with depression in the study results. Sleep quality and duration differ by sex and living arrangements [39, 40]; that is, females, older persons, and people with little education and low income are more likely to have poor sleep quality [40]. The average sleep duration of OWLA was 387.6 min (6.5 h) per day in our results; this is close to the average sleep duration of older adults [41]. Sleep patterns and depressive symptoms have non-linear relationships [42], and short or long sleep durations increase the risk of depression [43]. Although we did not focus on the mechanisms that link sleep and depression, and such mechanisms are indeed not fully understood [43], sleep is a reversible and correctable factor that decreases depression among OWLA. Thus, further studies that examine the causal relationship are needed, as well as more research on sleep duration, patterns, and timing, which could lower the risk of depression in this population. Moreover, non-pharmacological approaches and technology-assisted sleep interventions can improve sleep quality and mental well-being in OWLA.

Physical activity and sedentary behaviors were associated with HRQoL but were not related to depression. This is inconsistent with past studies, which suggested that physical inactivity is associated with depression [44]. However, a systematic review showed that it is difficult to find robust evidence of the effectiveness of physical activity for alleviating depression, which is consistent with our results [45]. The average time engaged in sedentary behaviors was 9.4 h in this study, which is much higher than older adults’ 5.3 h of self-reported sedentary behavior time revealed in a systematic review [46]. Regular physical activity and reduced sedentary behaviors are essential for active and healthy aging; therefore, the WHO proposed a global action plan on physical activity from 2018–2030 [47]. Additionally, the US Department of Health and Human Services suggested and distributed evidence-based physical activity guidelines by age group [48]. The guidelines mention that regular physical activity has a variety of benefits for older adults, including reducing risk of all-cause mortality, cardiovascular disease, cancer, and dementia. The guidelines also indicate that the benefits are not only improved sleep and quality of life but also decreased anxiety and falls [48]. The South Korean government set higher goals regarding physical activity through “Healthy People 2030.” These worldwide efforts emphasize the importance of maintaining regular physical activity and eliminating sedentary behavior. Our results provide evidence to support these policies. To ensure a safe environment for increasing physical activity and to enable situations that promote decreased sedentary behaviors among older adults, an approach based on a multidimensional system is required. Moreover, these strategies must target OWLA who lack supportive resources; interventions to improve the combination of sleep, physical activity, and sedentary behaviors will be effective for both depression and HRQoL in this population.

Equivalized household income as an enabling factor was only associated with HRQoL. Common factors related to depression and HRQoL were SHS, activity limitation, and perceived stress; these are all need factors. Self-rated health status has been tied to both depression and HRQoL in previous studies and is consistent with our findings [22, 49]. Relationships with other variables, such as activity limitation, stress, and income, are also supported by past reports [49, 50]. These common factors were also revealed in the cases that focused on OWLA in this study. In particular, the need factors showed large impacts on both result variables in our study. These findings were in the same context as previous studies that showed that the need factor is an important variable that influences outcome variables [51]. Thus, these common factors should be considered when preparing community-based services for older adults living alone in the public health sector.

In this study, Andersen’s model was employed to identify factors that affect depression and HRQoL in Korean OWLA, using nationally representative data. As is already known, Andersen’s model is multidimensional and explains how individual and contextual factors such as predisposing, enabling, and need factors affect health outcomes through health behaviors. Although the model has commonly been used to investigate the determinants of unmet health care needs, our study applied the model to better understand health outcomes among a vulnerable population. In particular, this study focused on several health behaviors, including sleep, physical activity, and sedentary behaviors, which confer benefits to older adults for many health conditions. The findings may be helpful for research stakeholders to understand factors related to OWLA’s depression and health-related quality of life, within a robust conceptual framework. Through this understanding, strategies may be generated to improve the health behaviors of OWLA to ensure a better life for them.

This study has several strengths and limitations. This study used a 4-year national survey sample that represents the Korean population, and all statistical results used sample weights; therefore, our results can be generalized. Furthermore, we adopted Andersen’s model as the research framework to develop a robust research design. This study also has several limitations. First, the major variables were investigated using self-reported questionnaires instead of objective measures such as sleep, physical activity, and sedentary behaviors. Second, several single-item questionnaires without validation were used. Third, c. 30.8% of the unweighted sample was excluded because of missing data; this could be a source of bias. Finally, although we relied on national data, the survey was conducted cross-sectionally; hence, causal relationships cannot be assumed.

## Conclusions

Health behaviors are crucial in maintaining healthy lives for OWLA. Our main findings showed that sleep was significantly associated with depression, while physical activity and sedentary behaviors were particularly related to HRQoL. Accurate and well-designed health interventions could promote adherence to necessary behavior change. Based on a comprehensive understanding of the characteristics of OWLA, healthcare providers need to deliver an approach rooted in a multidimensional system that incorporates interventions combined with strategies to increase physical activity, improve sleep quality, and decrease sedentary behaviors. This will be an effective way to improve the well-being of OWLA.

## Supplementary Information

**Additional file 1:Supplemental table 1.** Numbers of missing data (N = 1,147). **Supplemental table 2.** Survey questionnaires for included variables. **Supplemental table 3.** The association with health behaviors factors and depression, and health-related quality of life.

## Data Availability

The data are freely available and can be downloaded from the website for KNHANES of the KCDC.

## Abbreviations

EQ5D: EuroQol 5-Dimension
GPAQ: Global Physical Activity Questionnaire
HRQoL: Health-related quality of life
OWLA: Older women living alone
PHQ-9: Patient Health Questionaire-9
SHS: Subjective health status
WHO: World Health Organization
