# Supplementary material for: Sleep, physical activity, and sedentary behaviors as factors related to depression and health-related quality of life among older women living alone: a population-based study
Source: Eur Rev Aging Phys Act. 2023 Mar 13;20:6. doi: 10.1186/s11556-023-00314-7 (PMC10010020; doi:10.1186/s11556-023-00314-7)
Supplement: Supplementary file 1 — Additional file 1:Supplemental table 1. Numbers of missing data (N = 1,147). Supplemental table 2. Survey questionnaires for included variables. Supplemental table 3. The association with health behaviors factors and depression, and health-related quality of life. [file 11556_2023_314_MOESM1_ESM.docx]

**Supplemental table 1. Numbers of missing data (N = 1,147)**

| **Variables** | **N** |
| --- | --- |
| Depression (PHQ-9) | 208 |
| Health-related quality of life (EQ-5D) | 169 |
| ***Predisposing factors*** |  |
| Age | 0 |
| Education | 0 |
| ***Enabling factors*** |  |
| Equivalised income | 12 |
| Economic activity | 180 |
| ***Need factors*** |  |
| Multimorbidity | 147 |
| Subjective health status | 142 |
| Activity limitation | 153 |
| Perceived stress | 87 |
| ***Health behaviors*** |  |
| Sleep (mins/day) | 90 |
| Vigorous physical activity (METmin/week) | 167 |
| Moderate physical activity (METmin/week) | 167 |
| Sedentary behaviors (mins/day) | 258 |

**Supplemental table 2. Survey questionnaires for included variables.**

| **Health-related quality of life (EQ-5D)** |
| --- |
| By placing a tick in one box in each group below, please indicate which statements best describe your own health state today.  **1. Mobility**  □ I have no problems in walking about  □ I have some problems in walking about  □ I am confined to bed  **2. Self-care**  □ I have no problems with self-care  □ I have some problems washing or dressing myself  □ I am unable to wash or dress myself  **3. Usual activities**  □ I have no problems with performing my usual activities  □ I have some problems with performing my usual activities  □ I am unable to perform my usual activities  **4. Pain/discomfort**  □ I have no pain or discomfort  □ I have moderate pain or discomfort  □ I have extreme pain or discomfort  **5. Anxiety/depression**  □ I am not anxious or depressed  □ I am moderately anxious or depressed  □ I am extremely anxious or depressed |
| **Depression (PHQ-9)** |
| Over the last 2 weeks, how often have you been bothered by any of the following problems?  **1. Little interest or pleasure in doing things**  □ Not at all □ Several days □ More than half the days □ Nearly every day  **2. Feeling down, depressed, or hopeless**  □ Not at all □ Several days □ More than half the days □ Nearly every day  **3. Trouble falling or staying asleep, or sleeping too much**  □ Not at all □ Several days □ More than half the days □ Nearly every day  **4. Feeling tired or having little energy**  □ Not at all □ Several days □ More than half the days □ Nearly every day  **5. Poor appetite or overeating**  □ Not at all □ Several days □ More than half the days □ Nearly every day  **6. Feeling bad about yourself — or that you are a failure or have let yourself or your family down**  □ Not at all □ Several days □ More than half the days □ Nearly every day  **7. Trouble concentrating on things, such as reading the newspaper or watching television**  □ Not at all □ Several days □ More than half the days □ Nearly every day  **8. Moving or speaking so slowly that other people could have noticed? Or the opposite — being so fidgety or restless that you have been moving around a lot more than usual**  □ Not at all □ Several days □ More than half the days □ Nearly every day  **9. Thoughts that you would be better off dead or of hurting yourself in some way**  □ Not at all □ Several days □ More than half the days □ Nearly every day |
| **Age** |
| What is your the date of birth? |
| **Education** |
| What level of education do you have?  □ Less than elementary school □ Graduated secondary school □ Graduated high school □ Graduated university or higher |
| **Equivalised income** |
| What is your household income? (5 quintiles)  □ Low □ Lower- middle □ Middle □ Upper- middle □ High |
| **Economic activity** |
| Have you ever done paid work for one hour or more, or unpaid work for more than 18 hours, in the past week?  □ Yes □ No □ Not applicable |
| **Multimorbidity** |
| Do you currently have hypertension? (dyslipidemia, stroke, angina pectoris, myocardial infarction, osteoarthritis, osteoporosis, rheumatoid arthritis, pulmonary tuberculosis, asthma, diabetes, thyroid disease, gastric cancer, liver cancer, colon cancer, breast cancer, cervical cancer, lung cancer, thyroid cancer, other cancer 1, other cancer 2, depression, atopic dermatitis, allergic rhinitis, sinusitis, otitis media, renal failure, hepatitis B, hepatitis C, liver cirrhosis, gout)  □ I have |
| **Subjective health status** |
| How do you feel about your health?  □ Very poor □ Poor □ Fair □ Good □ Very good |
| **Activity limitation** |
| Do you currently have restrictions in daily life or social activities because of health problems or a physical or mental disability?  □ Yes □ No □ Not applicable |
| **Perceived stress** |
| Are you feeling stressed out in daily life?  □ Little □ A little bit □ A lot □ Very much □ Not applicable |
| **Sleep** |
| 1) How long do you take to sleep during the weekdays? ______ hours  2) How long do you take to sleep during the weekends or holidays? ______ hours |
| **Physical activity (GPAQ)** |
| Next I am going to ask you about the time you spend doing different types of physical activity in a typical week. Please answer these questions even if you do not consider yourself to be a physically active person.  In answering the following questions 'vigorous-intensity activities' are activities that require hard physical effort and cause large increases in breathing or heart rate, 'moderate-intensity activities' are activities that require moderate physical effort and cause small increases in breathing or heart rate.  **Activity at work**  Think first about the time you spend doing work. Think of work as the things that you have to do such as paid or unpaid work, study/training, household chores, harvesting food/crops, fishing or hunting for food, seeking employment.  1. Does your work involve vigorous-intensity activity that causes large increases in breathing or heart rate like *[carrying or lifting* *heavy loads, digging or construction work*] for at least 10 minutes continuously?  □ Yes □ No (*If No, go to 4)*  2. In a typical week, on how many days do you do vigorous-intensity activities as part of your work?  Number of days ________  3. How much time do you spend doing vigorous-intensity activities at work on a typical day?  Hours: minutes ________ : __________  4. Does your work involve moderate-intensity activity that causes small increases in breathing or heart rate such as brisk walking *[or carrying light loads*] for at least 10 minutes continuously?  □ Yes □ No (*If No, go to 7)*  5. In a typical week, on how many days do you do moderate-intensity activities as part of your work?  Number of days ________  6. How much time do you spend doing moderate-intensity activities at work on a typical day?  Hours: minutes ________ : __________  **Travel to and from places**  The next questions exclude the physical activities at work that you have already mentioned.  Now I would like to ask you about the usual way you travel to and from places.  For example, to work, for shopping, to market, to place of worship.  7. Do you walk or use a bicycle (*pedal cycle*) for at least 10 minutes continuously to get to and from places?  □ Yes □ No (*If No, go to 10)*  8. In a typical week, on how many days do you walk or bicycle for at least 10 minutes continuously to get to and from places?  Number of days ________  9. How much time do you spend walking or bicycling for travel on a typical day?  Hours: minutes ________ : __________  **Recreational activities**  The next questions exclude the work and transport activities that you have already mentioned.  Now I would like to ask you about sports, fitness and recreational activities (leisure).  10. Do you do any vigorous-intensity sports, fitness or recreational (*leisure*) activities that cause large increases in breathing or heart rate like [*running, football, climbing, swimming, basketball*] for at least 10 minutes continuously?  □ Yes □ No (*If No, go to 13)*  11. In a typical week, on how many days do you do vigorous-intensity sports, fitness or recreational (*leisure*) activities?  Number of days ________  12. How much time do you spend doing vigorous-intensity sports, fitness or recreational activities on a typical day?  Hours: minutes ________ : __________  13. Do you do any moderate-intensity sports, fitness or recreational *(leisure*) activities that causes a small increase in breathing or heart rate such as brisk walking, jogging, weight training, golf, dance sport for at least 10 minutes continuously?  □ Yes □ No (*If No, go to next)*  14. In a typical week, on how many days do you do moderate-intensity sports, fitness or recreational (*leisure*) activities?  Number of days ________  15. How much time do you spend doing moderate-intensity sports, fitness or recreational (*leisure*) activities on a typical day?  Hours: minutes ________ : __________ |
| **Sedentary behaviors** |
| The following question is about sitting or reclining at work, at home, getting to and from places, or with friends including time spent sitting at a desk, sitting with friends, traveling in car, bus, train, reading, playing cards or watching television, but do not include time spent sleeping.  How much time do you usually spend sitting or reclining on a typical day?  Hours: minutes ________ : __________ |

**Supplemental table 3. The association with health behaviors factors and depression, and health-related quality of life**

|  | **Depression** | | | | **Health-related quality of life** | | | |
| --- | --- | --- | --- | --- | --- | --- | --- | --- |
|  | **Model** | | | | **Model** | | | |
|  | **B** | **SE** | ***t*** | ***p*** | **B** | **SE** | ***t*** | ***p*** |
| **(Constant)** | 4.75 | 1.08 | 4.38 | <0.001 | 0.94 | 0.04 | 25.24 | <0.001 |
| **Health behaviors** |  |  |  |  |  |  |  |  |
| Sleep (mins/day) | -0.01 | 0.00 | -3.34 | <0.001 | 0.00 | 0.00 | 0.08 | 0.94 |
| Vigorous physical activity (MET min/week) | 0.00 | 0.00 | 0.03 | 0.97 | 0.00 | 0.00 | -3.12 | <0.001 |
| Moderate physical activity (MET min/week) | 0.00 | 0.00 | -0.61 | 0.54 | 0.00 | 0.00 | 3.55 | <0.001 |
| Sedentary behaviors (mins/day) | 0.00 | 0.00 | 4.10 | <0.001 | 0.00 | 0.00 | -6.92 | <0.001 |
| ***R^2^*** | 0.06 | | | | 0.10 | | | |
| **F *(P)*** | 10.85 (<0.001) | | | | 23.28 (<0.001) | | | |
